# Periodontitis, pathogenesis and progression: miRNA-mediated cellular responses to *Porphyromonas gingivalis*

**DOI:** 10.1080/20002297.2017.1333396

**Published:** 2017-06-12

**Authors:** Ingar Olsen, Sim K. Singhrao, Harald Osmundsen

**Affiliations:** ^a^ Department of Oral Biology, Faculty of Dentistry, University of Oslo, Oslo, Norway; ^b^ Dementia & Neurodegeneration Research Group, Faculty of Clinical and Biomedical Sciences, School of Dentistry, University of Central Lancashire, Preston, UK

**Keywords:** MiRNA, *P. gingivalis*, LPS, periodontitis, subversion of immunity, oral microbiome

## Abstract

*Porphyromonas gingivalis* is considered a keystone pathogen in periodontitis, a disease typically driven by dysbiosis of oral inflammophilic polymicrobial pathobionts. To combat infectious agents, the natural defense response of the host is to switch on inflammatory signaling cascades, whereby microRNA (miRNA) species serve as alternative genetic inhibitory transcriptional endpoints. miRNA profiles from diseased sites differ from those detected in disease-free tissues. miRNA profiles could therefore be harnessed as potential diagnostic/prognostic tools. The regulatory role of some miRNA species (miRNA-128, miRNA-146, miRNA-203, and miRNA-584) in the innate immune system suggests these molecular signatures also have potential in therapy. *P. gingivalis*–associated miRNAs are likely to influence the innate immune response, whereas its lipopolysaccharide may affect the nature of host miRNAs and their mRNA targets. This mini review discusses miRNA-dependent transcriptional and regulatory phenomena ensuing immune signaling cascade switch-on with development and progression of periodontitis initiated by *P. gingivalis* exposure.

## Introduction

MicroRNAs (miRNAs) constitute a family of small (~22 bases long), single-stranded, non-coding RNA molecules that selectively regulate translation of specific mRNAs. Numerous reports have identified the pathways of miRNA biogenesis [1], and a full description of this process is not within the scope of this mini review. It is important to remember that miRNAs are integral inhibitory signaling molecules generated from a primary transcript, which then causes an inhibitory function on a specific mRNA species. Briefly, their biogenesis involves primary miRNA (pri-miRNA) being transcribed by RNA polymerases II or III from either introns or intergenic regions [1]. Pri-miRNA is converted into precursor miRNA (pre-miRNA) by the microprocessor complex (MPC) unit, which consists of class 2 ribonuclease III enzyme (Drosha) and MPC subunit DGCR8 (Prasha). Pre-miRNAs are exported from the nucleus to the cytoplasm by exportin-5 where they undergo further cleavage by endoribonuclease (Dicer) into the miRNA/*miRNA duplex. In any one cell, usually one strand of the duplex remains as the mature miRNA and is incorporated into the miRNA-induced silencing complex (miRNAISC) with GW 182 proteins (recruited to the miRNA repressor complex in concert with Argonaute proteins) and Argonaute protein. The CCR4-NOT complex (a nine-subunit protein complex found widely in eukaryotes) recruited by GW182 causes translation inhibition of the mRNA and/or leads to de-adenylation of its poly (A) tail and subsequent degradation of the mRNA.

miRNA species appear to regulate diverse cellular functions (proliferation, metabolism, to tumorigenesis and adaptive immune pathways) in concert with their specific mRNAs. miRNAs function by exerting their sequence-specific binding to specific mRNAs, located primarily in the 3´ untranslated region (UTR). A key point here is that several miRNA species are capable of selectively targeting a single mRNA, whereas one miRNA may be targeted by several mRNAs. In this way, miRNAs can regulate the rate of mRNA translation. Therefore, changes in cellular levels of miRNAs are invariably associated with changes in the levels of target mRNA genes. This is likely to have a significant effect on downstream protein translation [1] because about 60% of all protein-coding genes are likely to have been miRNA targets [2].

miRNAs can affect both the innate and the adaptive immune systems [1]. Indeed, in diseased tissues (cardiovascular and Alzheimer’s diseases), substantial changes in the cellular levels of miRNAs have been described [3]. The ensuing miRNA profiles are potentially useful as bio/prognostic markers for diseased tissues [4], including periodontitis-affected periodontium [5]. Recently, it was described how *Porphyromonas gingivalis*, a keystone pathogen in periodontal disease, subverts both the innate and the adaptive immune responses, thereby facilitating its own survival and that of its radicalized co-species in the periodontal pocket [6–8]. The extent to which miRNAs are involved in this phenomenon remains under investigation, but differentially expressed genes encoding proteins associated with the innate immune responses appear to be associated with periodontitis-affected tissues [9,10].

The classical approach in studying miRNAs involves building their profiles from diseased and normal tissues. This facilitates the isolation and subsequent identification of differentially expressed miRNAs using appropriate software packages. The association of biological functions to differentially expressed miRNAs entails target mapping by using target analysis software, for example ShotStat 2.0 Target Analysis Software or Ingenuity® Pathways Analysis (IPA®) Software. Although an exploratory exercise, the concurrent profiling and identification of differentially expressed mRNAs is likely to facilitate greater significance in associations between miRNAs and mRNA targets. This also applies to likely functions of miRNAs in immune and stress responses in periodontal disease, as described by Kebschull and Papapanou [1] and Irwandi and Vacharaksa [11].

The aim of this mini review is twofold: first, to assess current knowledge as regards altered miRNA profiles found in *P. gingivalis*–mediated inflamed periodontium; and second, to determine possible functions of differentially expressed miRNAs in direct response to immune reactions that paradoxically facilitate bacterial survival and consequently promote pathogenesis/progression of periodontal disease.

**Figure 1.**
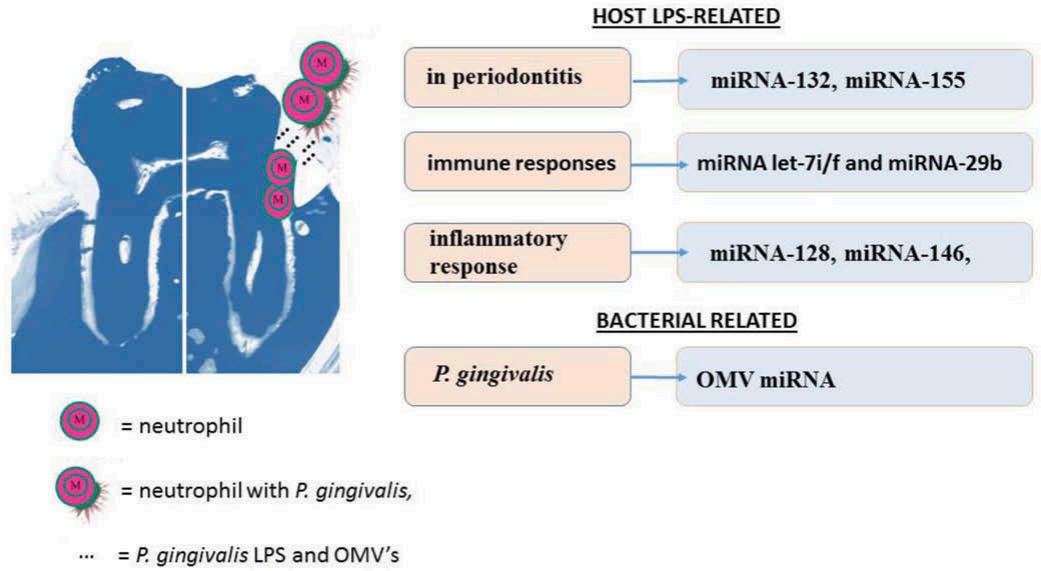
A molar tooth, invariably the target of periodontitis, shows one half with healthy peridontium (left) and the other with breached gingiva (right). Neutrophils are shown to represent their surveillance during health. Eventually, *Porphyromonas gingivalis* gains attachment and enters the cell to reach the subgingival niche. Here, it is able to survive, release lipopolysaccharide and outer membrane vesicles, and initiate periodontitis with its companion species (not shown). During disease initiation and its progression, several miRNA species are released that maintain a molecular communication with the host genes and self to promote its own survival and cause progressive damage to host tissues.

Key features of the innate immune response to *P. gingivalis* lipopolysaccharide (LPS, endotoxin) involves initiation of various signaling cascades within immune cells, for example neutrophils. Since neutrophils play key roles in maintaining periodontal health, most investigators have used the monocytic cell line THP-1 as a model to understand miRNA profiles following LPS exposure. During immune responses, numerous host proteins take part. These may include Toll-like receptors (TLRs), nuclear factor-kappaB (NF-κB), and p38 mitogen-activated protein kinase signaling (p38 MAPK) and others. The downstream effect of these pathways is in the liberation of cytokines and other related proteins to combat infection. miRNA species play an inherent transcriptionally important function as specific alternative genetic inhibitory transcriptional endpoints of signaling cascades. The host LPS-released miRNA species are listed in Figure 1.

## Possible functions of miRNA let-7i/f and miRNA-29b in periodontal immune responses

miRNA let-7i serves to inhibit translation of TLR 4 at the mRNA gene level [12]. This implies miRNA let-7i plays a pivotal role in the transcription of the immune receptor cell signaling system. However, this outcome may not apply to LPS from all oral pathogens. For example, levels of some miRNAs increase when cells are exposed to LPS from *Aggregatibacter actinomycetemcomitans* (miRNA let-7f) whereas *P. gingivalis* LPS-responsive TLRs (TLR 2, 4, or 7) can vary [12], and hence the LPS-responsive miRNA species differ (miRNA-29b). When investigating early miRNA responses of THP1-differentiated macrophages challenged with LPS from *A. actinomycetemcomitans* and *P. gingivalis*, Naqvi et al. [10] confirmed the detection of expected miRNA–mRNA interactions for *P. gingivalis* LPS-responsive miRNA 29b and *A. actinomycetemcomitans* miRNA let-7f using dual-luciferase assays. These experiments discovered miRNA-29b was targeted by mRNAs encoding interleukin (IL)-6Rα and interferon gamma inducible protein 30 (IFI30) genes. Let-7f appeared to target mRNAs encoding suppressor of cytokine signaling 4 (SOCS4) and thrombospondin-1 (TSP-1) [10]. SOCS4 and TSP-1 are host genes that play a role in mediating inflammation. The results by Naqvi et al. [10] provide evidence for the expected interplay between miRNA involvements in endotoxin mediated innate responses for the above genes.

## Effects of miRNA-128 on inflammatory response in periodontitis

Na et al. [13] hypothesized that miRNA-128 probably participates in the inflammatory response induced by *P. gingivalis* in periodontitis. To this end, they conducted experiments in which they exposed THP-1 and the oral squamous carcinoma CA9-22 cell lines to *P. gingivalis* [13]. They subsequently reported increased levels of miR-128 in the THP-1 and the CA9-22 cells [13]. Furthermore, by treating THP-1 cells with the miR-128 mimic, the expression levels of tumor necrosis factor-alpha (TNF-α) cytokine decreased significantly [13]. This result identified miRNAs with regulatory roles for TNF-α cytokine secretion. Downregulating TNF-α by miRNA-based therapy is of interest for treating diseases such as osteoarthritis because this cytokine plays a critical role in the pathogenesis of this debilitating disease [14]. Na et al. [13] also suggested that miR-128 mediates endotoxin tolerance through modulation of the p38 MAPK pathway. Thus, evolution has provided mechanisms for controlled inflammatory response in periodontitis that can compensate for the survival of the bacterium by providing sufficient sustenance and in turn maintain a chronic disease status by limiting damage to periodontal tissues: a win–win situation for the bacterium.

The p38 MAPK pathway signaling mediates inflammatory and stress responses. This pathway is critical in the regulation of levels of multiple pro-inflammatory cytokines (e.g. TNF-α, IL-1, IL-6, and IL-8), as well as for enzymes (cyclooxygenase and inducible nitric oxide synthase) involved during inflammation-mediated stress [15–17]. These enzymes maintain a balance in the host’s intrinsic and extrinsic oxidative stress. Given that periodontal disease has a polymicrobial dysbiotic etiology, it is not likely to be free from oxidative stress. Prolonged episodes of oxidative stress produce an excessive accumulation of reactive oxygen species, capable of inducing deleterious cellular and biomolecular damage to the host [18,19]. There is a paucity of information concerning the identity of miRNA profiles during acute phase reactive oxygen species generation in periodontal disease. As miRNAs bring about transcriptional endpoints of already switched-on signaling cascades (oxidative stress/miRNA regulation of nitric oxide synthase), their (miRNA) existence should be expected. p38 MAPK also regulates expression of matrix metalloproteinases [15,20], which are associated with destruction and remodeling of periodontal tissue. The increased levels of miRNA-128 may support a regulatory function during survival of *P. gingivalis* in the inflamed periodontium by muting the host’s inflammatory response that would otherwise be very harmful to the host tissues and to some extent also for the bacterium.

## Possible role for miRNA-132 in periodontitis

Park et al. [21] exposed THP-1-derived macrophages to *P. gingivalis* in order to study the expression of inflammatory miRNAs attempting to discover potential regulatory networks controlling innate immunity, also extending beyond TLR gene regulation. They found that *P. gingivalis* caused increased levels of miRNA-132 through primed TLR2/4 and NF-κB signaling, while a decreased level of miRNA-132 strongly suppressed TNF-α secretion. In this way, Park et al. [21] discovered expression of two transcription factor families (nuclear factor E2-related factor 2 [NFE2L2], and nuclear factor activated T cells [NFAT5]) that decreased in response to *P. gingivalis* infection. This result clearly suggests that *P. gingivalis* possesses the ability to regulate expression of inducible genes associated with immune responses aiming to eliminate this bacterium, as both the NFE2L2 and NFAT5 gene mRNAs are targets of miRNA-132. Transfection of THP-1 cells with the antagonist miRNA-132 restored levels of gene expression. These results support the conclusion that miRNA-132 has a role in the pathogenesis of periodontitis induced by *P. gingivalis* [21].

## Possible functions for miRNA-146 and miRNA-155 in periodontitis

Increased expression of miRNA-146a and miRNA-146a-5p from human gingival fibroblasts (HGFs) stimulated with *P. gingivalis* LPS was reported [22]. This report described elevated expression of both miRNA-146a and miRNA-146a-5p contributing to increased secretion of IL-1β, IL-6, and TNF-α [22]. These are classical host-mediated responses due to NF-κB-induced gene expression elicited by LPS. The involvement of miRNA-146 suggests that miRNA functions as an alternative signal to stimulate expression of genes associated with initiation of inflammation following *P. gingivalis* infection. Further evidence of miRNA functions during inflammation is derived from the finding that levels of IL-1 receptor associated kinase 1 (IRAK1) were increased while TNF receptor-associated factor 6 (TRAF6) levels remained unchanged. One explanation could be that miRNA-146a and miRNA-146b-5p are directly bound to 3´-UTR of IRAK1 mRNA, thereby halting expression of TRAF6 [22]. Alternatively, miRNA-146a and miRNA-146a-5p may act as negative regulators of the immune response by inhibiting expression of IRAK1 during periodontal microbial-initiated inflammation [22].

The miRNA-146-mediated IRAK1 response may also inhibit the inflammatory response in macrophages against LPS. Li et al. [23] reported that levels of miRNA-146a-5p were significantly elevated in macrophages 24 h after stimulation with a high dose of *P. gingivalis* LPS or an equivalent immunogen. The pro-inflammatory *P. gingivalis* LPS may participate in triggering dysregulation of the cassette transporters *ABCA1* and *ABCAG1* during periodontitis. These genes are also involved in the cholesterol transport system [24,25], suggesting possible adverse inflammatory effects in individuals suffering from dyslipidemia, for example individuals prone to atherosclerosis, stroke, and Alzheimer’s disease due to the shared apolipoprotein E, allelic variant 4 susceptibility gene (*APOE4*) inheritance. As a positive, macrophages transfected with miRNA-146a-5p mimics diminished the dysregulation of *ABCA1/G1* induced by *P. gingivalis* LPS through downregulation of IRAK1. This demonstrates a potential therapeutic potential of candidate miRNA for dyslipidemic subjects. Li et al. [23] suggested that in macrophages, miRNA-146a can operate as an inflammatory brake on LPS stimulation by decreasing the levels of two adapter proteins (IRAK1 and TRAF6) in an NF-κB-dependent manner [26], implying a strong therapeutic potential for miRNA-based therapies.

According to Krebshull and Papapanou [1], miRNA-146a is critical for enhancing the sensitivity of both TLR-like receptor and miRNA-155 for TLR in general. Also, altered levels of miRNA-146a affect expression levels of other miRNAs, implying inter-miRNA dependency for their regulation. Therefore, diverse biological effects of, for example, miRNA-155 may be due to changes in levels of other miRNAs, which are also regulated by miRNA-155, albeit directly or indirectly [1]. For example, miRNA-155 has a role in the regulation of adaptive immunity in periodontal disease [27], acting as a key modulator of the T-cell response [28].

*In vitro* studies performed to investigate if the *P. gingivalis* LPS affects levels of miRNA-146a in THP-1- and THP-1-derived macrophages entailed decreasing or increasing levels of miRNA-146a through transfection with a specific antagonist or precursor, respectively [29]. The results indicated only minor effects on cytokine production in macrophages that had been stimulated with *P. gingivalis* LPS while IRAK1 and TRAF6 remained unchanged [21,29]. In contrast, Nahid et al. [30] studying periodontitis as a polyinfection in the ApoE^–^^/^^–^ mouse model, found *P. gingivalis* LPS stimulated THP-1 that were transfected with miRNA-146a antagonist prior to stimulation led to about a 90% decrease in the level of miRNA-146a, followed by an increase in TNF-α response to TLR ligands. Since these investigations tried correlating miRNA-146a changes with decreased levels of IRAK1 and TRAF6, this has given rise to discrepancies. While these discrepancies could be resolved by accounting for differences in experimental conditions, an alternative explanation is a paucity of information regarding unknown mRNAs also being targeted by miRNA-146a. Thus, any significance of data from the above studies will become available once the role of miRNA-146a in cells stimulated with TLR ligands is established. Others have proposed [29] a broader function for a single miRNA (e.g. miRNA-146) because it can target several mRNAs [31].

Jiang et al. [32] reported that *P. gingivalis* LPS (10 µg/mL) increased the expression of TLR2, TLR4, and miRNA-146a in human periodontal ligament cells (HPDLCs). At 1 µg/mL LPS, *P. gingivalis* induced TLR2, miRNA-146a, and NF-κB p65 nuclear activity and enhanced cytokine secretion but without affecting TLR4. However, upregulation of miRNA-146a was inhibitory to both anti-TLR2/4 monoclonal antibodies (mAb) [32]. Rather surprisingly, after blocking with the aforementioned mAbs, miRNA-146a, TLR2, TLR4, NF-κB p65 nuclear activity, and pro-inflammatory cytokines increased. These results demonstrate subtleties in the overlapping function of mAbs and suggest patient-tailored therapies may be useful with dual-acting immunoglobulins. Further research is required to understand the mechanisms by which these mAbs exert their synergistic effect. Experiments using HPDLCs stimulated with *P. gingivalis* LPS exposed to a miRNA-146a mimic showed that the levels of miRNA-146a, TLR2, TLR4, NF-κB p65 nuclear activity, and proinflammatory cytokine parameters decreased significantly. This led to the conclusion that miRNA-146a functions as a negative feedback regulator by downregulating proinflammatory cytokine secretion and blocking TLR signaling in HPDLCs after stimulation with *P. gingivalis* LPS.

## Possible role for miRNA-203 in *P. gingivalis* pathogenesis

It is understood that miRNA-203 levels increase post exposure of human gingival epithelial cells (GECs) to live, invasive *P. gingivalis*. This has led to the notion that activity of the cytokine signaling pathway regulation is a miRNA-203-mediated event [33]. To this end, GECs infected with *P. gingivalis* revealed an increased level of miRNA-203, whereas levels of putative mRNA targets of miRNA-203, that is, suppressor of cytokine signaling 3 (SOCS3) and SOCS6, decreased (0.2 and 0.5 relative to controls) [33]. SOCS3 regulates cytokine signals, which modulate receptor activation of NF-κB ligand (RANKL)-mediated osteoclastogenesis [34], and SOCS6 has an important role modulating mitochondrial dynamics and subsequent apoptotic events [35]. The results indicate a role for miRNA-203 in the pathogenesis of *P. gingivalis* by contributing to regulation of host-cell immune responses in a manner promoting bacterial persistence.

## *P. gingivalis*–dependent increase in the level of miRNA-584 inhibits anti-inflammatory effects

miRNA-584 targets the mRNA encoding the lactoferrin receptor (LfR). This was determined by exposing the immortalized human GEC line OBA-9 to *P. gingivalis*. The results demonstrated a threefold increase in miRNA-584 and suppression of the LfR at both the mRNA and protein level [36]. The transfection of OBA-9 cells with a miRNA antagonist for miRNA-584 recovered *P. gingivalis–*dependent suppression of LfR supporting the aforementioned observation. A similar result was observed with *P. gingivalis*–stimulated OBA-9 cells for the human lactoferrin (hLf), which markedly downregulated the synthesis of IL-8. This suggested that the *P. gingivalis*–dependent increase in the level of miRNA-584 observed in OBA-9 cells diminished the anti-inflammatory response by decreasing the level of LfR. This is another plausible transcriptional therapeutic target for mitigating inflammation.

## Miscellaneous miRNAS act as regulators in *P. gingivalis* LPS-induced periodontitis

Du et al. [37] examined miRNA profiles in periodontal ligament cells (PDCLs) treated with *P. gingivalis* LPS. Using miRNA microarrays, they found 50 different miRNA species that were differentially expressed, of which 22 exhibited increased levels and 28 exhibited decreased levels of expression. Levels of expression of seven randomly selected miRNAs from each population (miRNA-21-5p, −498, and −548-5p – increased expression) and (miRNA-495-3p, −539-5p, −34-3p, and −7a-2-3p – decreased expression) were confirmed using quantitative reverse transcription polymerase chain reaction. Target analysis of these miRNAs using the miRWalk database suggested significant associations to cellular functions related to LPS-induced periodontitis. These included the TLR signaling pathway, the cAMP-signaling pathway, the transforming growth factor-beta signaling pathway, the MAPK signaling pathway, and other related pathways. These results support the notion that several miRNA species may selectively target a single mRNA and *vice versa*. The mechanism by which this genetic stimulation is switched off during LPS-induced periodontitis remains to be defined.

## Outer membrane vesicles from *P. gingivalis* containing miRNA

Bacteria communicate between themselves and with other bacteria *via* signaling molecules, including LPS, proteins, and nucleic acids found in the membrane or lumen of outer membrane vesicles (OMVs) [38]. A typical example is OMVs from *P. gingivalis* [39]. Recently, a novel class of miRNAs was characterized in *P. gingivalis, A. actinomycetemcomitans*, and *Treponema denticola* OMVs. This indicates that some bacteria harbor miRNA-like molecules that are resilient to degradation by RNAses found in the host’s body fluids (e.g. serum and saliva). These miRNAs are suggested to function as bacterial signaling molecules that can be transferred both to other bacteria and into host cells *via* OMVs, facilitating the modulation of host immune responses [40].

## Concluding remarks

The relationship between *P. gingivalis* and miRNAs at present is vague. The fact that most of the reports reviewed here originated from *in vitro* studies is a reflection of this new and developing field of research. Many of the studies discussed are from cultured cell lines exposed to LPS. Data from LPS experiments do not necessarily translate to the data with live bacteria, although both approaches have merits. However, it is clear that miRNA profiles of healthy and diseased periodontal tissues are significantly different [9,10]. The miRNA approach may therefore prove to be fruitful in future studies of periodontal disease, especially in relation to the keystone pathogen *P. gingivalis*.

Current data suggest a role for miRNA in the virulence of *P. gingivalis*, particularly contributing to modulation of host-cell immune responses in a manner promoting bacterial survival, and progressively reducing the host’s protective function. It is rather surprising that some of the aforementioned miRNAs are also associated with *P. gingivalis* itself [40] while others (miRNA-128, miRNA-146, miRNA-203, and miRNA-584) are host derived. It is noteworthy that the bacterium-associated miRNAs are likely to influence the innate immune response against *P. gingivalis*, whereas LPS from this bacterium may affect the levels of the host’s miRNA–mRNA interactions. These miRNA-dependent effects may supplement other forms of deception exerted by *P. gingivalis* serving to subvert innate and adaptive immune responses. It is most important to remember that the oral microbiome consists of hundreds of microbial species, each expressing various pro-inflammatory factors likely to be involved in the development of periodontitis. Therefore, the outcome in resolving disease in the context of miRNAs based biomarkers and therapy should envisage contributions from polymicrobial infections.

It is recognized that *P. gingivalis* requires some inflammatory response to acquire iron and proteins for survival and growth. Thus, low-grade ‘tick-over’ inflammation is favorable for this bacterium and to some degree for the host. However, the miRNAs associated with chronic inflammatory diseases should be targets for therapeutic interventions and, where possible, also serve as diagnostic biomarkers. Further research will contribute to a better understanding of the functions and the mode of miRNAs working in the pathogenesis of periodontitis to determine whether and how miRNAs can be harnessed for the diagnosis and therapy of periodontitis.
